# Global and Regional Burden of Crohn’s Disease (1990-2023): A Global Burden of Disease Study Analysis

**DOI:** 10.7759/cureus.113724

**Published:** 2026-07-31

**Authors:** Omar Abureesh, Mohammad Abu-Shaban, Yousef Yousef, Sanad Elshebli, Mutaz Alsaleh, Mohammad Yousef, Abdullah Abureesh, Ahmad Maswadeh, Asad Ur Rahman

**Affiliations:** 1 Internal Medicine, Staten Island University Hospital, Staten Island, USA; 2 Internal Medicine, Southeast Georgia Health System, Brunswick, USA; 3 Trauma and Orthopedics, Buckinghamshire Healthcare NHS Trust, Buckinghamshire, GBR; 4 Obstetrics and Gynecology, University Hospitals Birmingham NHS Foundation Trust, Birmingham, GBR; 5 Internal Medicine, University of Jordan, Amman, JOR; 6 Trauma and Orthopedics, West Middlesex University Hospital, London, GBR; 7 Gastroenterology, Cleveland Clinic Florida, Weston, USA

**Keywords:** biological therapies, crohn’s disease, global burden of disease, inflammatory bowel disease, prevalence

## Abstract

Background: Crohn's disease (CD) is an inflammatory bowel disease (IBD) that has evolved into a global health concern, with a rising cumulative burden that makes periodic, up-to-date quantification essential for health-system planning. In recent decades, its incidence has stabilized in early-industrialized regions while rising rapidly in newly industrialized ones, resulting in an increasing cumulative disease burden in high-income regions. This epidemiological transition necessitates precise burden estimation to inform strategic health policies.

Methods: The study was conducted using openly available modeled estimates from the Global Burden of Disease (GBD) 2023 study, in an independent effort to analyze the regional and global burden of CD from 1990 to 2023. This analysis was conducted in accordance with the Guidelines for Accurate and Transparent Health Estimates Reporting. Main outcome measures were disability-adjusted life years (DALYs), years of life lost (YLLs), and years lived with disability (YLDs), expressed as absolute values and age-standardized rates per 100,000 people across 21 GBD regions. The contributions of population growth and age-standardized rate change to the observed DALY were examined after being separated using a two-component decomposition, in addition to the temporal patterns across the whole period. YLLs and YLDs were calculated for each year, and for 2023, male-to-female DALY rate ratios were observed over time.

Results: Between 1990 and 2023, aggregate DALYs attributable to CD rose from approximately 271,700 to 414,300 (+52.5%), while the age-standardized DALY rate declined modestly (-5.6%). Decomposition indicated that this increase was driven predominantly by demographic change rather than by rising age-standardized risk. Eight of the 21 GBD regions nevertheless showed genuine rate-driven increases, most prominently in Tropical Latin America and in Sub-Saharan Africa. Burden remained highly heterogeneous, with a greater than 20-fold difference between the highest age-standardized rate (Australasia, 19.70 per 100,000; 95% UI: 13.86-26.89) and the lowest (East Asia, 0.95; 95% UI: 0.67-1.30). The YLD share of DALYs rose from 49.2% to 55.4%, indicating a shift toward disability-dominated burden. Male-to-female rate ratios varied widely across regions, with female predominance concentrated in Sub-Saharan Africa. In the North Africa and Middle East region, the age-standardized rate fell by 12.3% while absolute burden nonetheless grew.

Conclusion: The global burden of Crohn's has increased since 1990, driven principally by population expansion rather than worsening disease risk. However, genuine rate-driven increases in Tropical Latin America and Sub-Saharan Africa signal epidemiological transitions demanding targeted surveillance and resource allocation. The shift toward disability predominant burden underscores a growing need for long-term disease management globally. These findings are limited to regional aggregates for CD only; country-level analyses incorporating ulcerative colitis data are needed to fully characterize the global IBD burden.

## Introduction

Crohn’s disease (CD) is a chronic inflammatory intestinal disease, first described as regional ileitis by Crohn et al. [1] in a case series presented at American Medical Association annual meeting in 1932, and it is one of the major entities within the spectrum of inflammatory bowel diseases (IBDs) and is a chronic inflammatory disease of the gastrointestinal tract that might lead to progressive bowel damage and disability [2].

CD develops when genetic, environmental, and microbial factors interact to trigger an abnormal, persistent immune response in the intestinal mucosa [3]. Patients commonly present with abdominal pain, chronic diarrhea, weight loss, fatigue, and rectal bleeding. Progressive inflammation can lead to complications like strictures and fistulas, while widespread extraintestinal manifestations significantly increase morbidity and reduce quality of life [4].

CD exerts a substantial negative impact on health-related quality of life (HRQoL), affecting physical, psychological, and social well-being. The chronic and relapsing nature of the disease, coupled with symptoms such as abdominal pain, diarrhea, fatigue, and weight loss, often interferes with daily activities, employment, and interpersonal relationships. Studies consistently demonstrate that patients with active disease report significantly poorer quality of life than those in remission, with disease activity emerging as one of the strongest determinants of HRQoL. Furthermore, frequent relapses, hospitalizations, work disability, extraintestinal manifestations, and psychological distress are associated with worse outcomes. Female sex and previous disease-related complications may further impair quality of life. Conversely, effective biological therapies and sustained disease control have been linked to meaningful improvements in HRQoL. Recent evidence also suggests that nutritional status and muscle strength are important contributors to both physical and mental health domains, highlighting the multifactorial nature of quality-of-life impairment in CD [5,6].

Accurately quantifying the burden of CD requires measures that go beyond prevalence and mortality. Disability-adjusted life years (DALYs) provide a comprehensive assessment of disease burden by combining years of life lost (YLLs) due to premature mortality and years lived with disability (YLDs) resulting from nonfatal health outcomes. While CD is associated with relatively low mortality compared with many other chronic diseases, its recurrent symptoms, complications, need for long-term treatment, and substantial impact on physical, psychological, and social functioning contribute significantly to disability. Consequently, YLDs represent a major component of the overall burden, whereas YLLs capture the mortality-related impact of severe disease and its complications. Evaluating DALYs, YLLs, and YLDs together enables a more comprehensive understanding of the population-level consequences of CD, facilitates comparisons across regions and time periods, and provides essential evidence for healthcare planning, resource allocation, and public health policy development [7,8].

The Global Burden of Disease (GBD) Study systematically quantifies mortality, disability, and health loss for diseases and injuries worldwide, using standardized measures such as DALYs, YLLs, and YLDs to enable comparison across populations and over time [8,9]. It thereby supports the evaluation of temporal, geographic, and sex-specific patterns of burden to inform health planning and priority setting.

Although substantial progress has been made in understanding the epidemiology of CD, important knowledge gaps remain. Many previous studies have primarily focused on incidence and prevalence trends, whereas comparatively fewer investigations have evaluated the overall burden of CD using DALYs, YLDs, and YLLs as comprehensive measures of health loss. Furthermore, despite the availability of GBD estimates, limited research has comprehensively examined long-term burden trends spanning more than three decades and assessed changes from 1990 onward using the most recent datasets. Crucially, the GBD 2023 update marks the historic first time that CD and ulcerative colitis have been disaggregated and estimated as independent causes of disease burden. In addition, the relative contributions of demographic factors, including population growth and population aging, compared with epidemiological changes, have not been extensively evaluated in CD burden studies. These evidence gaps are especially relevant in the North Africa and Middle East region, where IBD incidence and prevalence have increased substantially in recent decades, but comprehensive burden assessments remain relatively scarce. Therefore, an updated analysis using the latest GBD 2023 estimates is warranted to provide a contemporary evaluation of the burden of CD across populations and regions. Such analyses can support evidence-based healthcare planning, resource allocation, disease surveillance, and future research prioritization.

Accordingly, this study had four prespecified objectives: 1) to quantify the burden of CD as DALYs, YLLs, and YLDs, expressed both as absolute counts and as age-standardized rates per 100,000 population, across all 21 GBD regions for each year from 1990 to 2023; 2) to quantify the relative contributions of demographic change and of change in age-standardized rates to the observed change in absolute DALYs over the same period and regions; 3) to characterize the changing composition of burden by estimating the proportion of DALYs attributable to YLDs vs. YLLs annually across the study period, and to quantify sex differences by computing male-to-female age-standardized DALY rate ratios for 2023 in each region; and 4) to examine the North Africa and Middle East region in depth, benchmarked against Western Europe, high-income North America and East Asia as comparator regions. Each objective is specific in its outcome and population, measurable against published GBD 2023 estimates, achievable using publicly available data, relevant to health-system planning for a chronic disabling condition, and time-bound to the period 1990-2023.

## Materials and methods

Data source and study design

This work is an independent secondary analysis of publicly available data on the global, regional, and national burden of CD during 1990-2023, in accordance with the Guidelines for Accurate and Transparent Health Estimates Reporting (GATHER) [10]. The analyzed data were obtained from the Institute for Health Metrics and Evaluation (IHME) Global Health Data Exchange and the IHME GBD Results Tool (Institute for Health Metrics and Evaluation, Seattle, WA; vizhub.healthdata.org/gbd-results). The performance of this study was independent of the GBD collaboration, and all analyses were conducted exclusively by the authoring team. GATHER checklist is listed in the Appendix. The underlying GBD 2023 estimates are generated by the IHME using a Bayesian framework, in which nonfatal outcomes (prevalence, YLDs) are modeled with DisMod-MR 2.1 (Institute for Health Metrics and Evaluation, Seattle, WA) and cause-specific mortality with the Cause of Death Ensemble model. These models were run by the GBD collaboration; the present study analyzes their published outputs and fits no model of its own.

Data extraction and handling

Estimates were extracted from the GBD Results Tool using the GBD 2023 release. The extraction specified cause (CD), measures (DALYs, YLLs, YLDs), metrics (number and rate), locations (21 GBD regions), sexes (both, male, female), ages (all ages and age-standardized), and years (1990-2023 inclusive).

Age-standardized rates were downloaded directly from the GBD Results Tool as published by the IHME; they were not recalculated by the authors, and no alternative standard population was applied. Analyses were conducted on the published point estimates, which correspond to the posterior means of the GBD 2023 modeling process. Draw-level data were not downloaded, and the 1,000 individual draws were therefore not used.

Uncertainty intervals (UIs) are reported as published for all directly extracted quantities, namely age-standardized DALY rates in the Results section and in Table 1. Because draw-level data were not used, uncertainty was not propagated through derived analyses. The temporal decomposition, the YLD and YLL proportions of DALYs, and the male-to-female rate ratios were therefore computed from point estimates only and are presented without UIs. This is a deliberate limitation of the present analysis and is noted in the Discussion section; a draw-level implementation would permit formal uncertainty propagation and is identified as a priority for future work. The extracted series were complete: estimates were available for all 21 GBD regions, all sexes, and all years from 1990 to 2023, with no missing values. No imputation, interpolation, or exclusion of observations was therefore required at any stage.

**Table 1:** Age-standardized DALY rates with 95% uncertainty intervals, absolute DALY numbers, YLD proportions, and percent change for all 21 GBD regions Regions are sorted by percent change in age-standardized rate (descending). Rates per 100,000 people (both genders, age-standardized). Absolute DALYs (all ages) are shown by numbers UI: uncertainty interval; YLD: years lived with a disability; DALY: disability-adjusted life year; GBD: Global Burden of Disease

Region	DALY rate 1990 (95% UI)	DALY rate 2023 (95% UI)	% Change	YLD % 1990	YLD % 2023	DALYs (n) 1990	DALYs (n) 2023
Tropical Latin America	4.37 (2.58-6.71)	6.28 (4.37-8.76)	+43.6%	48.8%	56.7%	2,879	12,588
Southern Africa	4.42 (2.60-6.79)	5.26 (3.37-7.69)	+19.0%	50.1%	57.6%	1,572	2,850
Andean Latin America	2.65 (1.61-4.04)	3.12 (2.04-4.58)	+17.9%	49.5%	57.1%	1,363	2,032
Western Sub-Saharan Africa	1.21 (0.71-1.89)	1.38 (0.89-2.02)	+14.0%	51.7%	59.3%	2,783	16,979
Southeast Asia	2.63 (1.59-4.00)	2.97 (1.99-4.19)	+12.8%	50.1%	57.7%	5,895	10,224
Eastern Sub-Saharan Africa	1.48 (0.87-2.31)	1.63 (1.04-2.38)	+10.3%	51.2%	58.8%	2,136	4,393
Australasia	16.54 (11.17-23.21)	19.70 (13.86-26.89)	+19.1%	47.5%	54.6%	2,218	6,161
Central Latin America	4.16 (2.51-6.30)	4.39 (3.01-6.18)	+5.5%	48.9%	56.9%	5,218	9,385
North Africa and Middle East	5.03 (3.48-6.97)	4.41 (3.03-6.18)	-12.3%	49.1%	56.4%	13,289	27,163
High-income North America	18.56 (12.75-25.85)	19.40 (13.90-26.07)	+4.5%	47.2%	54.4%	25,448	56,549
South Asia	2.50 (1.53-3.77)	2.30 (1.59-3.16)	-8.6%	50.3%	57.9%	40,440	76,987
Caribbean	5.19 (3.14-7.88)	4.70 (3.21-6.65)	-9.5%	48.9%	56.5%	1,431	2,185
Southern Latin America	7.27 (4.53-10.74)	6.51 (4.60-8.91)	-10.5%	47.8%	55.0%	3,237	4,391
Western Europe	15.75 (10.89-21.83)	13.32 (9.59-18.10)	-15.4%	47.3%	54.5%	56,406	71,860
Oceania	4.22 (2.49-6.45)	3.35 (2.22-4.79)	-20.6%	50.6%	58.0%	353	416
East Asia	1.29 (0.83-1.87)	0.95 (0.67-1.30)	-26.4%	50.7%	58.3%	15,629	20,088
High-income Asia Pacific	10.48 (7.06-14.74)	7.65 (5.51-10.36)	-27.0%	47.7%	55.0%	10,042	11,201
Central Sub-Saharan Africa	1.55 (0.90-2.41)	1.08 (0.70-1.58)	-30.3%	51.3%	58.9%	946	1,636
Eastern Europe	12.57 (8.57-17.52)	8.64 (6.23-11.72)	-31.3%	47.5%	54.8%	30,100	25,597
Central Asia	10.10 (6.70-14.39)	6.95 (4.93-9.58)	-31.2%	48.0%	55.2%	6,399	7,252
Central Europe	9.84 (6.64-13.83)	6.59 (4.74-8.94)	-33.0%	47.6%	54.8%	7,640	8,054

Scope and coverage

GBD cause code: B.2.3.1 for CD was used to accumulate data across 21 GBD super-regions and regional aggregates, covering both sexes, all ages, and age-standardized measures during the period of 1990-2023. Analyses were conducted at the level of the 21 GBD regions. Country-level GBD 2023 estimates are publicly available through the IHME Results Tool, and regional aggregation was adopted as a deliberate analytic choice rather than as a consequence of any restriction on data access. For a comparatively low-prevalence condition such as CD, country-level estimates in data-sparse settings depend heavily on predictive covariates and on borrowing of strength from regional priors within DisMod-MR 2.1, and are correspondingly less stable. Regional aggregates provide a more robust basis for the 33-year comparative and decomposition analyses undertaken here. Country-level analysis remains an important extension of this work and is identified as such in the Discussion section.

Outcome measures

In the present study, the primary measures were DALYs, the sum of YLLs and YLDs, expressed as absolute counts and as age-standardized rates per 100,000 population. YLLs were computed as deaths multiplied by the residual life expectancy at age of death using the GBD 2023 standard life table. YLDs were computed as prevalence multiplied by GBD disability weights for mild, moderate, and severe CD. All estimates are reported with 95% UIs, as per GBD’s 1,000-draw approach across modeling stages.

Statistical analyses

Trend Analysis

To analyze age-standardized DALY rates per 100,000 population, results over the full 1990-2023 time series per GBD region were plotted. The absolute DALY numbers for all ages were summed across GBD superregions to derive aggregate estimates on a global scale. The relative change per region was calculated using the percentage age-standardized rates between 1990 and 2023. Trends are summarized by the relative change in age-standardized rate between 1990 and 2023; estimated annual percentage change modeling and forward projection were not undertaken.

Burden Decomposition (YLL vs. YLD)

To characterize the relative contributions of premature mortality vs. disability compared with the total DALY burden, the proportion of YLL or YLD attributable DALYs was computed per year and per region. Time-based shifts in the YLD and the YLL proportions were then analyzed to identify any transition between mortality-dominated and disability dominated burden characteristics.

Temporal Decomposition

To account for population growth and compare it with true epidemiological rate changes, we utilized a two-component decomposition, decomposing the total DALY change per region as follows:

$\Delta \mathrm{DALY}_{\mathrm{total}} {=} \text{Population effect} {+} \text{Rate effect}$

where $\text{Demographic effect} {=} \left(\mathrm{Population}_{2023} {-} \mathrm{Population}_{1990}\right) {\times} \frac{\text{Mean rate}}{100,000}$ and $\text{Rate effect} {=} \left(\mathrm{Rate}_{2023} {-} \mathrm{Rate}_{1990}\right) {\times} \frac{\text{Mean population}}{100,000}$. Because each term is evaluated at the mean of the other factor, this is the symmetric two-factor decomposition of Kitagawa, which is exact rather than approximate: the cross-terms cancel identically, so that the two components sum precisely to the observed change and no residual term arises. This identity was confirmed numerically for all 21 GBD regions, where residuals did not exceed floating-point precision (below 10^-^⁹ DALYs). The decomposition is exact in this algebraic sense; it remains first-order with respect to the underlying factors, in that it partitions change into a demographic and a rate component without further separating population growth from population aging. 

Sex-Disaggregated Analysis

To characterize sex disparities, male-to-female DALY rate ratios were computed for 2023 and plotted across regions.

MENA Regional Focus

Due to the significance of the Middle East and North Africa (MENA) region with regard to the burden of IBD, we examined this region in depth. DALY rates, YLL/YLD composition, sex ratios, and temporal trends were compared with Western Europe, High-income North America, and East Asia as comparator regions.

Software

All statistical analyses and visualizations were performed using Python 3.14.4 (pandas, NumPy, matplotlib, seaborn; Python Software Foundation, Fredericksburg, VA) and R 4.6 (tidyverse, ggplot2, patchwork, ggrepel; R Foundation for Statistical Computing, Vienna, Austria).

## Results

Global and regional burden of CD (1990-2023)

**Figure 1:**
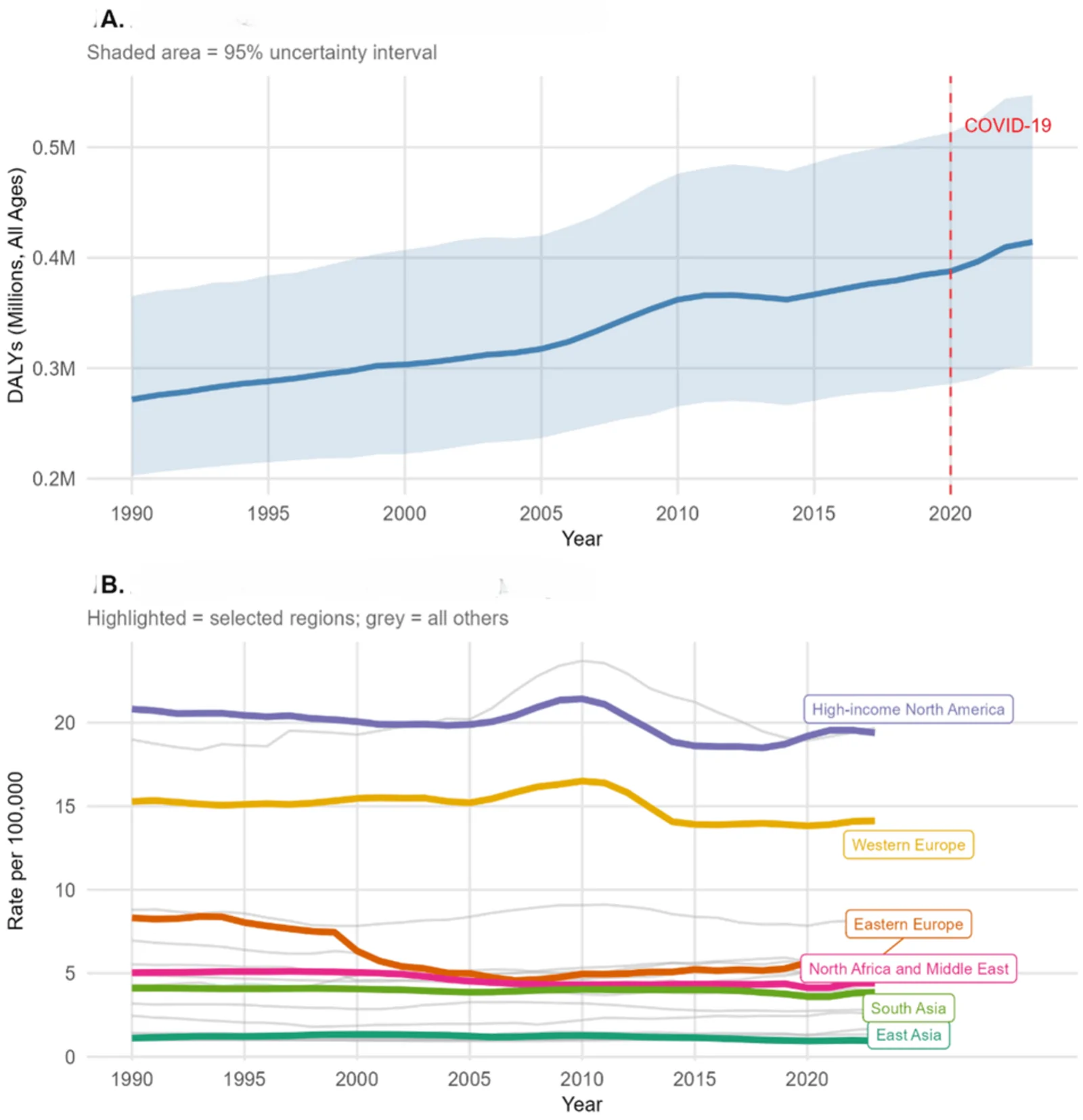
Global and regional DALY trends of Crohn's disease (1990-2023) (A) Aggregate absolute DALY burden summed across GBD regions (all ages, both genders). (B) Age-standardized DALY rates per 100,000 by GBD region. Shaded band: 95% uncertainty interval. Dashed red line: COVID-19 pandemic onset (2020). Highlighted lines: selected regions; gray lines: all others DALY: disability-adjusted life year; GBD: Global Burden of Disease

In brief, the absolute burden of CD rose substantially between 1990 and 2023, while its age-standardized rate declined slightly, a divergence driven by demographic change; the detailed estimates follow. The aggregate DALY burden attributable to CD between 1990 and 2023 increased substantially across GBD regions, rising from approximately 271,700 DALYs in 1990 to 414,300 DALYs in 2023, representing an absolute increase of +52.5% (Figure 1A). Despite the absolute burden that had risen, the mean age-standardized DALY rate had declined from 5.75 to 5.43 per 100,000 population (-5.6%) (Figure 1B). To add, marked geographic heterogeneity was observed throughout the study period. In 2023, Australasia recorded the highest age-standardized DALY rate (19.70 per 100,000; 95% UI: 13.86-26.89), followed closely by high-income North America (19.40 per 100,000; 95% UI: 13.90-26.07). In contrast, East Asia reported the lowest burden at 0.95 per 100,000 (95% UI: 0.67-1.30). Eastern Europe was the only GBD region (1/21, 4.8%) to record an absolute decline in DALYs over the study period.

Decomposition of DALY trends

**Figure 2:**
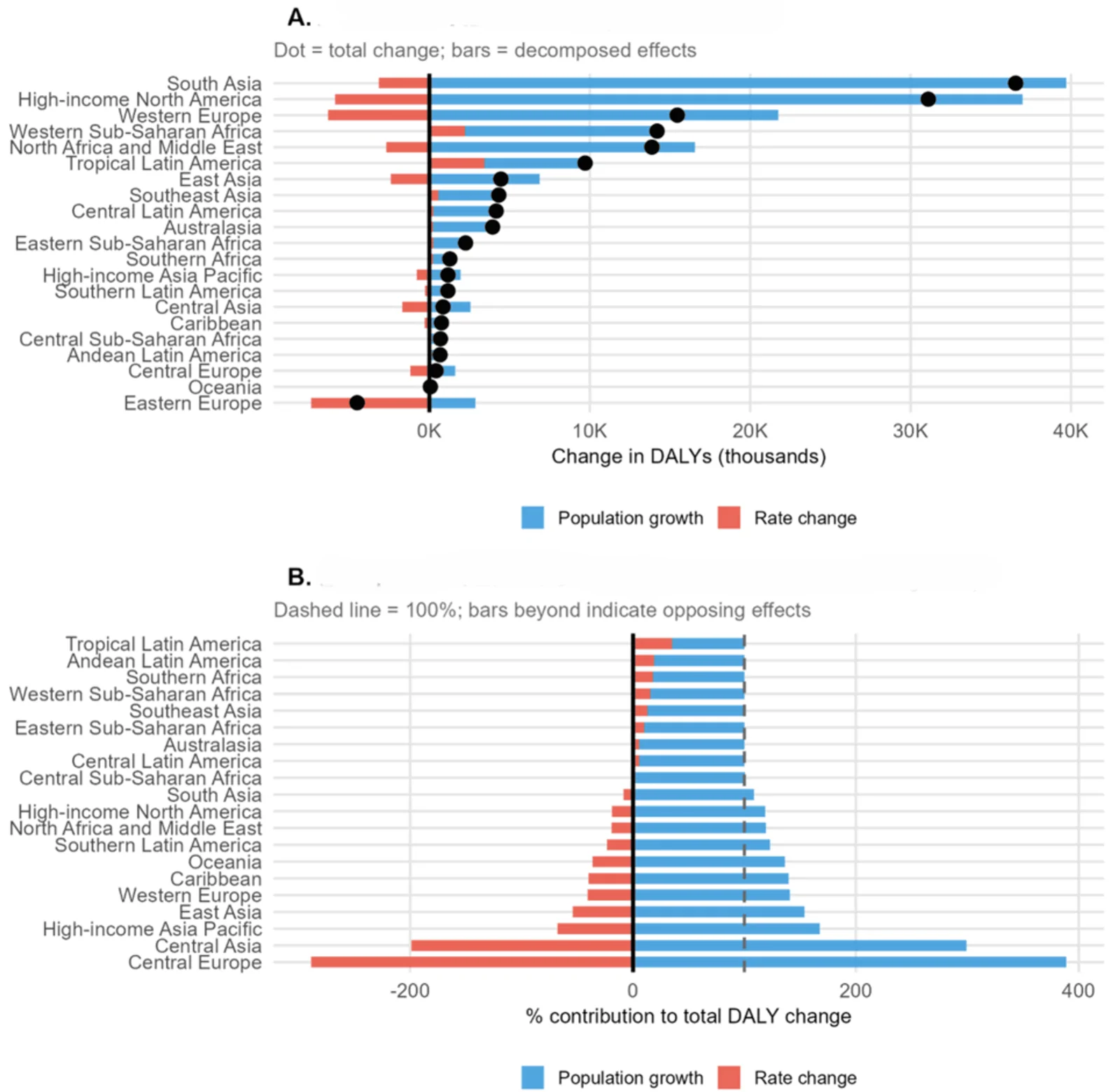
Temporal decomposition of DALY change (1990-2023) Crohn's disease (A) Absolute DALY change decomposed into population growth effect (blue) and rate change effect (red) for each GBD region. Black dot represents total DALY change. (B) Proportional contribution of each effect (positive-delta regions only). Dashed line represents 100%; bars extending beyond indicate partially opposing effects DALY: disability-adjusted life year; GBD: Global Burden of Disease

To distinguish the contributions of demographic change from true epidemiological changes, a decomposition of the 1990-2023 DALY change for each region was performed, splitting the data into a population growth effect and an age-standardized rate change effect (Figure 2, Table 1).

Across GBD regions, the net increase of 142,567 DALYs was mostly attributable to demographic change (population growth and aging combined) (+167,374 DALYs; 117.4% of total change), which was offset partially by a favorable decline in age-standardized rates (-24,807 DALYs; -17.4%). This pattern, in which demographic change outweighed improvements in age-adjusted risk, was consistent in most regions. Eight of the 21 GBD regions (8/21, 38.1%) recorded a positive rate-change effect, indicating that CD burden worsened independently of demographic factors. Rate-driven increases were most pronounced in Tropical Latin America (rate effect: +35.3% of total DALY change), Andean Latin America (+19.0%), Southern Africa (+17.8%), and Western Sub-Saharan Africa (+15.7%). In contrast, Central Europe and Central Asia showed rate-driven decreases in which the two components nearly canceled, leaving small net changes (approximately +414 and approximately +853 DALYs); proportional contributions are not reported for these regions, as expressing a component as a percentage of a near-zero net change yields uninformative values exceeding 100% in magnitude.

Composition of burden: YLLs vs. YLDs

**Figure 3:**
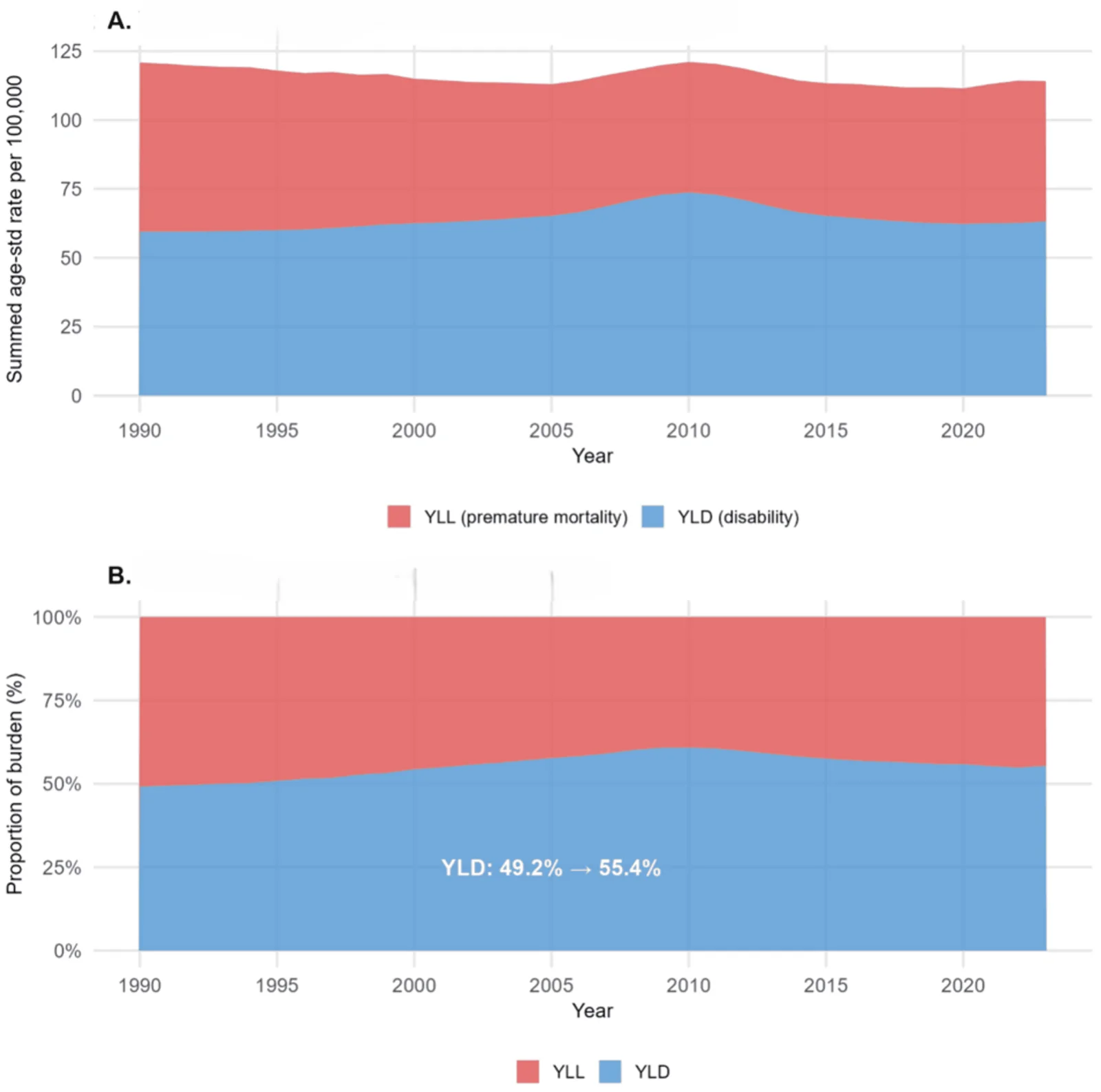
YLL vs. YLD decomposition: Crohn's disease (1990-2023) (A) Absolute age-standardized contribution of YLLs (red) and YLDs (blue) summed across GBD regions. (B) Proportional contribution of YLLs and YLDs to total DALY burden over time YLL: years of life lost; YLD: years lived with disability; DALY: disability-adjusted life year; GBD: Global Burden of Disease

A shift in DALY burden over the study period was observed (Figure 3). In 1990, YLLs and YLDs contributed almost equally, with YLDs accounting for 49.2% of DALYs. By 2023, the YLD proportion became 55.4%.

Sex-disaggregated analysis

**Figure 4:**
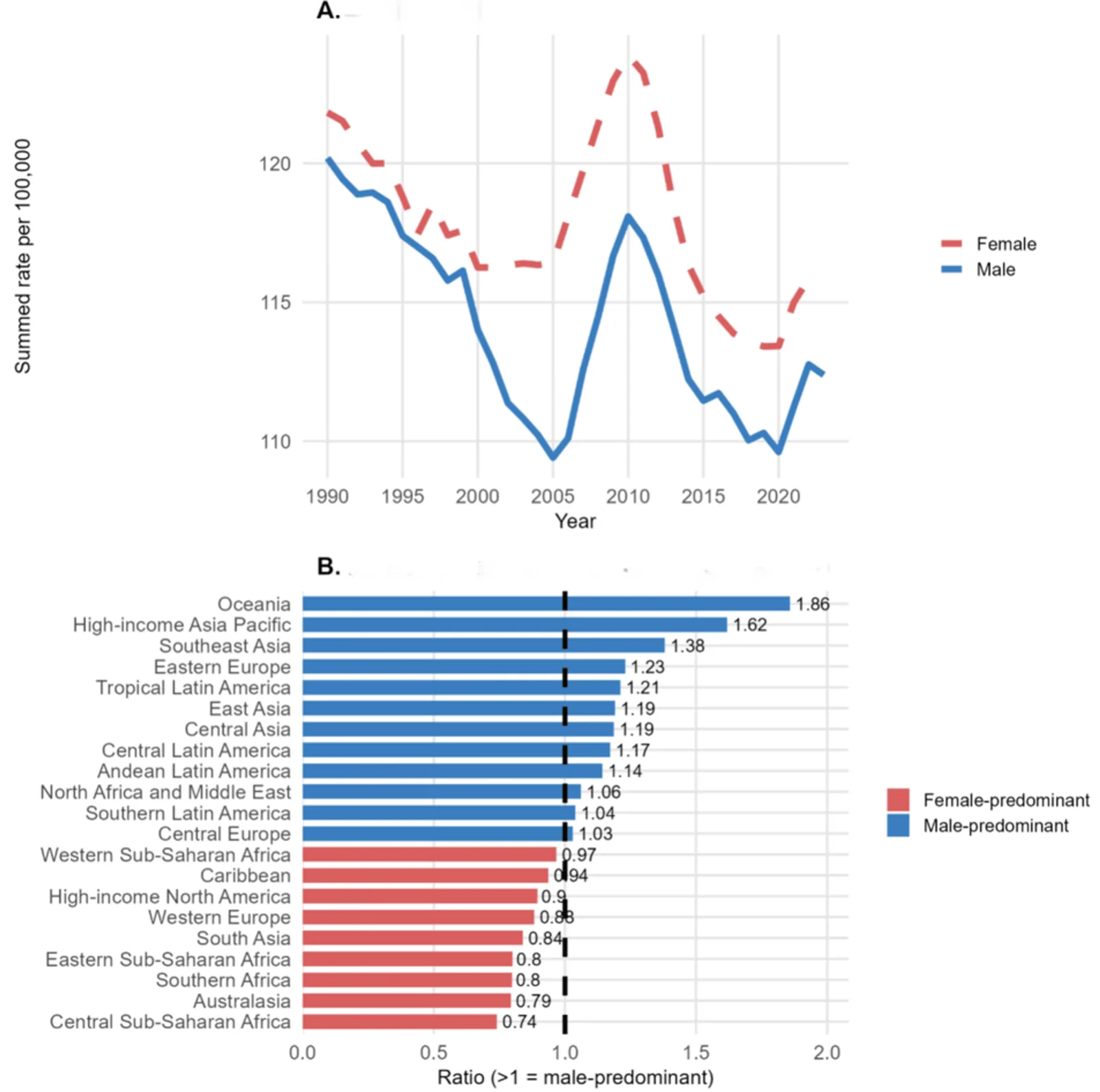
Sex-disaggregated DALY analysis: Crohn's disease (1990-2023) (A) Aggregate male and female age-standardized DALY rates over time. (B) Male-to-female DALY rate ratio by GBD region in 2023. Red: female-predominant, blue: male-predominant, Dashed line: parity (ratio = 1) DALY: disability-adjusted life year; GBD: Global Burden of Disease

Male patients had a higher age-standardized DALY rate in most GBD regions throughout the study period, though female predominance was observed in nine of the 21 regions (Figure 4). The male-to-female DALY rate ratio ranged from 0.74 (Central Sub-Saharan Africa) to 1.86 (Oceania) in the year of 2023. Nine of the 21 GBD regions (9/21, 42.9%) showed female predominance, with Sub-Saharan African regions exhibiting the highest female predominance.

Regional analysis: MENA region

**Figure 5:**
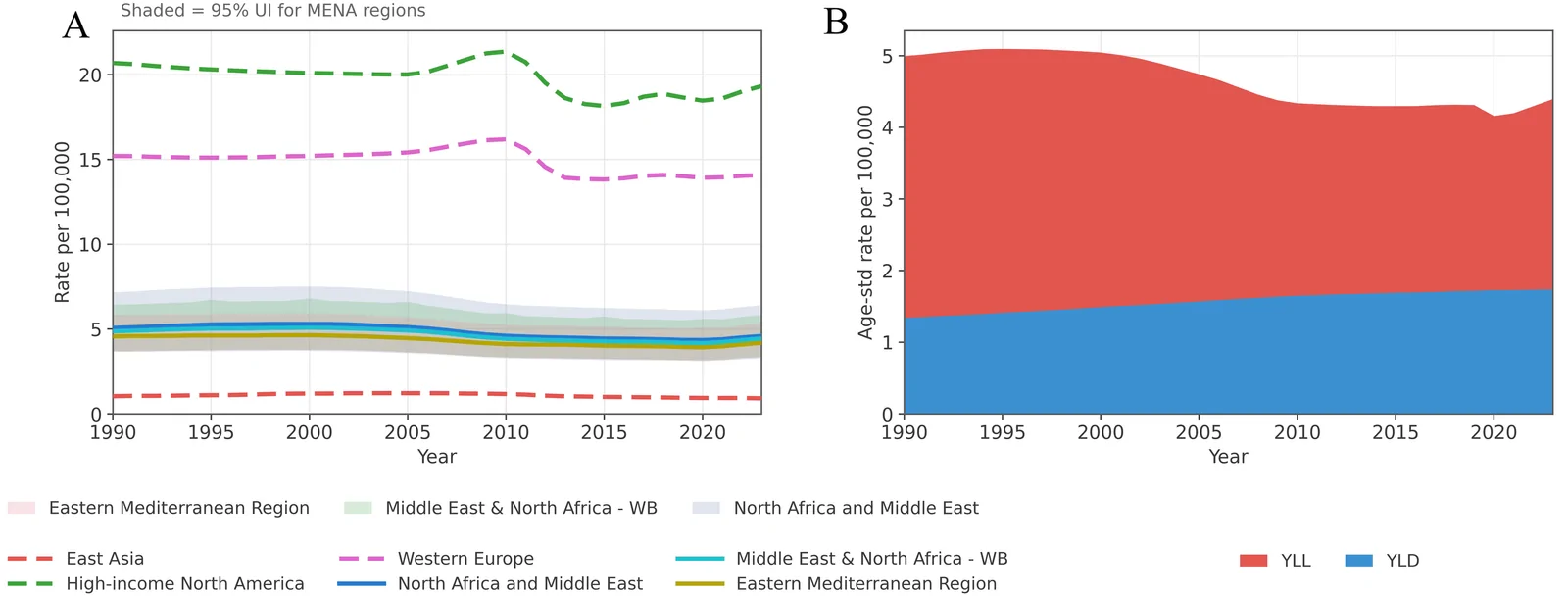
MENA-focused analysis: Crohn's disease (1990-2023) (A) Age-standardized DALY rates for MENA regions vs. comparators. (B) YLL vs. YLD decomposition for North Africa and the Middle East. Shaded bands represent 95% UIs; Gold border represents the MENA region MENA: Middle East and North Africa; YLL: years of life lost; YLD: years lived with disability; DALY: disability-adjusted life year; UI: uncertainty interval; WB: World Bank

**Figure 6:**
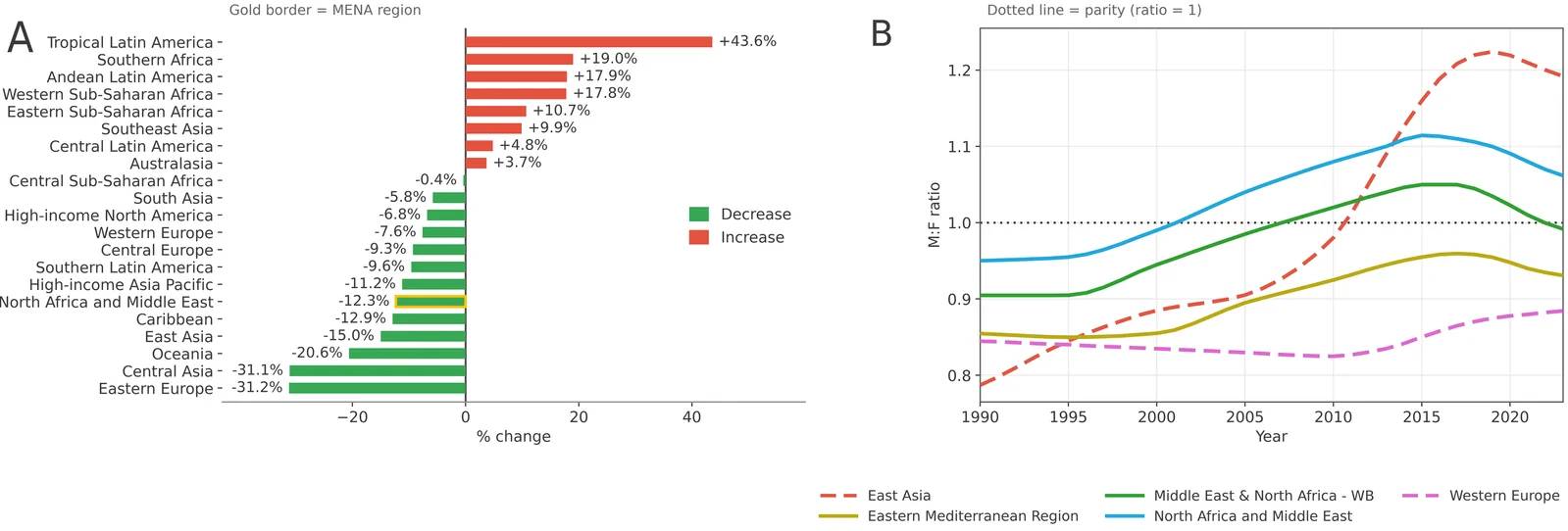
Age-standardized DALY trends: MENA vs. world regions (A) Percent change in age-standardized DALY rate (1990-2023) across all GBD regions. (B) Male-to-Female DALY rate ratio trend for MENA vs. comparator regions. Shaded bands represent 95% UIs; Gold border represents the MENA region MENA: Middle East and North Africa; DALY: disability-adjusted life year; GBD: Global Burden of Disease; UI: uncertainty interval; WB: World Bank

The MENA region is of epidemiological interest. During the study period, the age-standardized DALY rate declined from 5.03 per 100,000 in 1990 to 4.41 per 100,000 in 2023 (95% UI: 3.03-6.18; -12.3%), which is a trajectory consistent with trends in Western European comparators (Figure 5). However, absolute DALY burden had risen by 13,874 DALYs, which is the fifth largest absolute increase among GBD regions, overwhelmingly driven by population growth (population effect: +16,542 DALYs) rather than changes in age-standardized risk (rate effect: -2,668 DALYs). The decomposition of CD burden within the region reveals a gradual shift from YLL-dominated to YLD-dominated burden, in coherence with the global pattern (Figure 5). The male-to-female DALY rate ratio remained relatively stable, indicating persistent male predominance (Figure 6).

## Discussion

An update on the worldwide and regional burden of CD from 1990 to 2023 is provided by this secondary analysis of GBD 2023 estimates. The epidemiological transition of CD is similar to more general trends observed in other immune-mediated and noncommunicable diseases, where Westernization and industrialization cause an initial increase in prevalence that then plateaus as disease control improves [11,12]. Our results support three main themes: the disease's developing clinical presentation toward a chronic, disability-managed condition, the ongoing change in the epicenter of CD burden, and the predominant role of demographic factors in determining absolute case numbers. While the mean age-standardized DALY rate decreased slightly (from 5.75 to 5.43 per 100,000; -5.6%), the overall burden increased significantly over the research period, with aggregate DALYs rising by almost 52.5% (from ~271,700 to ~414,300).

A significant epidemiological disparity can be seen in the difference between a 52.5% increase in absolute DALYs and a little 5.6% decrease in the age-standardized rate. This is made clear by the two-component decomposition: population growth contributed +167,374 DALYs (117.4% of the change) to the net rise of 142,567 DALYs, which was somewhat offset by a favorable rate effect of -24,807 DALYs (-17.4%). Therefore, population growth and aging are significantly more responsible for the growing load than any inherent rise in risk at the individual level. This trend has been observed in the literature on IBD and other chronic illnesses; thus, it is not specific to CD [13-15]. A recent GBD-derived analysis has likewise attributed the great majority of the rising IBD burden, particularly among older adults, to population growth rather than rising rates [5]. The practical corollary is that the encouraging improvement in per-capita risk is being overwhelmed by demographic momentum: health system planning cannot rely on per-capita risk reduction alone but must proactively adapt to a larger absolute number of individuals living with CD, especially older patients who present distinct management challenges [14].

Our findings are directionally consistent with earlier GBD cycles, though not numerically comparable with them. Analyzing GBD 2019, Wang et al. [3] reported a fall in the global age-standardized DALY rate for IBD from 27.20 to 20.15 per 100,000 (1990-2019) alongside a 29.98% rise in absolute DALYs, and GBD 2021 describes a comparable decline from 21.55 to 18.07 per 100,000, the same divergence between falling rates and rising absolute burden that we observe for CD. Direct comparison is precluded, however, because every prior cycle modeled IBD as a single cause, whereas GBD 2023 is the first to estimate CD and ulcerative colitis independently; cross-cycle comparison is therefore qualitative.

A striking feature of our findings is the marked geographic heterogeneity in disease burden. In 2023, Australasia recorded the highest age-standardized DALY rate (19.70 per 100,000; 95% UI: 13.86-26.89), closely followed by high-income North America (19.40 per 100,000), whereas East Asia recorded the lowest (0.95 per 100,000); a greater than 20-fold difference. This gradient reaffirms the strong association between CD and a Westernized environment and is consistent with the observation that historically high-incidence, early-industrialized regions in North America, Europe, and Oceania continue to carry the heaviest prevalence-driven burden, having entered the “compounding prevalence” stage of IBD evolution in which incidence has plateaued while prevalence rises steadily as the patient population ages [11,12]. Mechanistically, the Westernization hypothesis posits that a Western diet high in animal fat, refined sugars, and ultraprocessed foods but low in fiber, together with reduced microbial exposure, increased antibiotic use, and smoking, perturbs the gut microbiome and promotes a dysbiosis state that triggers CD in genetically susceptible individuals [9]. The comparatively low rate in East Asia should be interpreted with caution, as it may partly reflect differences in diagnostic ascertainment and case definition rather than a true absence of disease, given the documented rapid acceleration of IBD incidence across Asia in recent decades [16,17]. It should be emphasized that no dietary, microbial, behavioral, or socioeconomic exposures were analyzed in this study; these mechanisms are offered only as hypotheses consistent with the observed geographic gradient and are not tested here.

Eight regions exhibited a genuine, rate-driven increase in burden, a worsening independent of demographic expansion. The most pronounced rate effects occurred in Tropical Latin America (+35.3% of total DALY change), Andean Latin America (+19.0%), Southern Africa (+17.8%), and Western Sub-Saharan Africa (+15.7%). This pattern fits the “acceleration in incidence” phase of the four-stage epidemiological model for IBD, characterized by a rapid rise in new diagnoses as regions industrialize and lifestyles shift [11,12]. Supporting population-based data are concordant: the incidence of CD in Brazil rose from 0.08 per 100,000 person-years in 1988 to 5.5 per 100,000 person-years in 2015, while in Colombia CD incidence increased from 0.48 to 0.83 per 100,000 between 2017 and 2019. These rate-driven increases therefore likely represent early epidemiological transitions rather than statistical noise, and signal a pressing need for enhanced surveillance, diagnostic capacity, specialist services, and preventative public-health messaging in health systems not historically configured to manage chronic IBD. Eastern Europe was the only GBD region to record an absolute decline in DALYs over the study period; by contrast, the very large rate-driven decreases modeled in Central Europe and Central Asia (rate effects of -288.8% and -199.0% of their regional change, respectively) should be interpreted cautiously, as their extreme proportional magnitude may partly reflect modeling assumptions and data sparsity rather than solely real-world clinical gains [16,18].

The composition of the DALY burden shifted meaningfully over the study period: the proportion attributable to YLDs rose from 49.2% in 1990 to 55.4% in 2023, marking a transition from a mortality-influenced to a disability-dominated burden profile. This is consistent with the relatively low mortality of CD combined with improving survival. The introduction and increasingly early use of immunomodulators and biologic agents such as antitumor necrosis factor therapies, vedolizumab, and Ustekinumab has improved clinical remission rates and reduced hospitalization and surgery, transforming CD into a chronic condition managed over decades rather than a frequently fatal one [19,20].

The magnitude of this disability burden is now well recognized. A systematic review found that approximately 25.8% of IBD patients experience depression and 21.2% anxiety, with rates particularly elevated in CD, and psychological distress is independently associated with higher disease activity, increased healthcare utilization, and treatment escalation [20]. Such findings demand a parallel shift in healthcare priorities: health systems must move beyond a purely biomedical, treat-to-target approach aimed at endoscopic healing to incorporate comprehensive, patient-centered models integrating mental-health support, nutritional counseling, and rehabilitation [21,22]. The dominant unmet need is increasingly the sustained, multidisciplinary stewardship of a chronic condition, with disability prevention and preservation of quality of life as central goals.

Male predominance was the global norm, but the male-to-female DALY rate ratio varied widely from 0.74 in Central Sub-Saharan Africa to 1.86 in Oceania in 2023, and nine of the 21 GBD regions (9/21, 42.9%) exhibited female predominance, concentrated in Sub-Saharan Africa. This heterogeneity is well documented but incompletely understood: a female excess in CD has been reported in adult North American and European populations, whereas a male excess has been described in several Asian and Middle Eastern populations. For example, female predominance has been observed for CD in Northern France, while in Iran male CD patients had higher odds of active disease than female patients. Potential drivers include sex-specific effects of smoking, hormonal influences on immune function, and differences in health-seeking behavior. The pronounced female predominance in Sub-Saharan African regions is particularly intriguing and may be genuine or reflect diagnostic biases, and differential ascertainment between the sexes cannot be excluded. This area remains critically underresearched and warrants dedicated, sex-stratified epidemiological and mechanistic study.

The MENA region presented a distinct profile that acts as a bellwether for the global transition of CD. Its age-standardized DALY rate declined from 5.03 to 4.41 per 100,000 (-12.3%), broadly consistent with Western European comparators, yet its absolute burden grew by 13,874 DALYs, the fifth largest absolute increase among all GBD regions. Decomposition attributed this growth overwhelmingly to population expansion (population effect +16,542 DALYs) rather than rising age-standardized risk (rate effect -2,668 DALYs). The decline in age-standardized rates appears at first to conflict with earlier reports of rising incidence and prevalence in the region through 2019, but this may be reconciled by our longer observation window and the possibility that the rate decline is a more recent phenomenon reflecting improving disease management; the consistent message across analyses is the overwhelming role of demographic pressure. Notably, the wide UI around the 2023 estimate (3.03-6.18) reflects the scarcity of high-quality, population-based data, which derives largely from single-country registries such as the Iranian Registry of Crohn’s and Colitis and nationwide Iranian surveys [22]. Strengthening national registries and surveillance infrastructure is an urgent prerequisite for generating more precise future burden estimates and for planning within often underresourced health systems.

When combined, these results have a number of implications for health planning. Even in areas where age-standardized risk is declining, the rising absolute burden indicates a growing demand for IBD services due to demographic momentum. This calls for proactive investments in diagnostic capacity, access to biologic therapy, and specialized care, especially in regions like Sub-Saharan Africa and Latin America that are truly experiencing rate-driven increases. The transition to a burden that is disability-predominant highlights the importance of long-term, comprehensive chronic disease management and quality-of-life assistance. Establishing reliable disease registries should be seen as a fundamental step for evidence-based resource allocation in data-poor regions like MENA and Sub-Saharan Africa.

Strengths and limitations

The use of standardized, globally comparable GBD methodology with adherence to the GATHER, a 33-year observation window, a decomposition approach that clearly distinguishes between demographic and epidemiological drivers, and supplementary sex-disaggregated and MENA-focused analyses are the main advantages of this analysis.

There are a few restrictions to be aware of. First, there are significant regional differences in the quality and accessibility of primary data. In many low- and middle-income settings, especially in Sub-Saharan Africa and parts of Asia, a lack of disease registries and a lack of diagnostic infrastructure likely result in underascertainment, so low recorded rates may partially reflect underdiagnosis rather than genuinely low disease frequency. Second, residual uncertainty persists, as evidenced by the large UIs, even though GBD uses advanced tools like DisMod-MR 2.1 to estimate burden in data-sparse regions. Third, the study is limited to regional aggregates and may, therefore, mask substantial within-region variability. Country-level estimates are publicly available, and their analysis represents a natural and important extension of this work. Fourth, because the study only looks at CD, it may not fully reflect the burden of IBD. Additionally, in settings without comprehensive endoscopic and histological evaluation, it is still feasible to misclassify ulcerative colitis as CD. Fifth, the two-component decomposition separates demographic change from change in age-standardized rates but does not separate population growth from population aging; the demographic component therefore represents their combined contribution, and no claim is made regarding their relative magnitude. A three-component decomposition separating population growth, aging, and epidemiological change, as used in contemporary GBD analyses, would resolve this and is the principal extension we intend to pursue. Sixth, population denominators used in the decomposition were reconstructed from all-age DALY counts and age-standardized rates rather than taken from official GBD population estimates. Because age-standardized rates are normalized to a reference age structure, this reconstruction systematically overstates population change, and the demographic component of the decomposition is inflated as a result. The direction of the principal finding, that demographic change rather than rising age-standardized risk accounts for most of the increase in absolute burden, is robust to this limitation, since world population grew by roughly half over a period in which absolute DALYs grew by 52.5%. The precise magnitudes of the two components, however, and particularly the estimated offsetting contribution of falling age-standardized rates, should be regarded as approximate.

Future research should include ulcerative colitis to characterize the entire IBD burden, explore country-level analysis to address within-region variance, and use age-period-cohort and projection modeling to predict future needs. To reduce the data uncertainty that currently limits burden estimation in these locations, it is necessary to conduct mechanistic and epidemiological research on regional heterogeneity in sex patterning and to continue investing in national IBD registries, particularly throughout MENA and Sub-Saharan Africa.

## Conclusions

The worldwide burden of CD is increasingly marked by a divergence between a slight drop in age-standardized rates and an increasing total number of cases caused by population increase and aging, according to this study of GBD 2023 data. The most alarming trends are the rate-driven increases seen in portions of Latin America and Sub-Saharan Africa, indicating a new front in the global epidemiology of CD, even while high-income regions continue to shoulder the most burden. The necessity for health systems to transition toward long-term chronic disease management with an emphasis on disability prevention and patient outcomes is highlighted by the change toward a YLD-dominant burden. The MENA region serves as a sentinel for the intricate interaction between better clinical results and overwhelming demographic pressure, and the variation in sex disparities between regions shows a significant knowledge gap requiring targeted inquiry. Overall, under current demographic trends and without a decline in age-standardized rates large enough to offset them, the absolute societal and healthcare burden of CD is likely to continue rising, though no formal projection was undertaken here; this necessitates proactive, context-specific, and resource-aware global health policies, even if real progress has been made in reducing individual-level risk.

## Appendices

**Table 2:** Guidelines for accurate and transparent health estimates reporting CD: Crohn's disease; DALYs: disability-adjusted life years; GATHER: Guidelines for Accurate and Transparent Health Estimates Reporting; GBD: Global Burden of Disease; GHDx: Global Health Data Exchange; IHME: Institute for Health Metrics and Evaluation; LMICs: low- and middle-income countries; MENA: Middle East and North Africa; UI: uncertainty interval; YLDs: years lived with disability; YLLs: years of life lost; UC: ulcerative colitis

S. no.	GATHER checklist item	How the manuscript addresses it	Reported in
Objectives and funding			
1	Define the indicator(s), populations (age, sex, geographies), and time period(s) for which estimates were made	Indicators: DALYs, YLLs, YLDs (absolute counts and age-standardized rates per 100,000). Populations: both sexes (and sex-disaggregated), all ages and age-standardized, across 21 GBD regions/super-regions. Period: 1990-2023	Title; Abstract (Methods); Methods - “Scope and Coverage” and “Outcome Measures.”
2	List the funding sources for the work	Authors received no funding; explicitly stated	“Funding Statement” (end matter)
Data inputs - sources synthesized in the study			
3	Describe how data were identified and how they were accessed	Estimates retrieved from the IHME GHDx and GBD Results Tool (vizhub.healthdata.org/gbd-results); exact query parameters (cause, measures, metrics, age, sex, location, year) are listed.	Methods - “Data Source and Study Design”; “Data Availability Statement.”
4	Specify inclusion and exclusion criteria; identify all ad hoc exclusions	Included: GBD cause code B.2.3.1 (Crohn’s disease), 21 GBD regions, both sexes, all ages and age-standardized, 1990-2023. Excluded: country-level estimates. These are publicly available but were not analyzed; regional aggregation was a prespecified scope decision	Methods - “Scope and Coverage”; Discussion - limitations
5	Provide information on all included data sources and their main characteristics (reference, population, collection method, years, sex/age range, diagnostic/measurement method)	The synthesized input is the modeled GBD 2023 estimate set. Its characteristics are specified: population (21 GBD regions, both sexes, all ages), period (1990-2023), and case definition (GBD cause code; disability weights for mild/moderate/severe CD). Underlying primary-source inputs are documented within the GBD 2023 study and cited via the GBD methods reference	Methods - “Outcome Measures”; GBD methods reference
6	Identify and describe categories of input data with potentially important biases	Regional differences in primary-data quality, absence of registries/diagnostic infrastructure in many LMICs, likely underascertainment/underdiagnosis, and possible misclassification of ulcerative colitis as CD are described	Discussion - “Strengths and limitations”
Data inputs - inputs used but not synthesized			
7	Describe and give sources for any other data inputs	Auxiliary inputs identified: GBD 2023 standard life table (YLLs), GBD disability weights (YLDs), and GBD population estimates/denominators (rates and decomposition)	Methods - “Outcome Measures” and “Temporal Decomposition.”
Data inputs - for all data inputs			
8	Provide all data inputs in an efficiently extractable file format, or a contact for data that cannot be shared	Input estimates are publicly available and downloadable in tabular form from the GHDx Results Tool; the documented query parameters allow exact reproduction of the extraction	“Data Availability Statement”
Data analysis			
9	Provide a conceptual overview of the data analysis method	A narrative overview of the analytic workflow is given: trend analysis, YLL/YLD decomposition, two-component (population vs rate) temporal decomposition, sex-disaggregated analysis, and MENA focus	Methods - “Statistical Analyses”
10	Provide a detailed description of all analysis steps, including mathematical formulas	Steps are described with formulas: YLL = deaths × residual life expectancy; YLD = prevalence × disability weight; population-effect and rate-effect equations for the decomposition; method for implied population denominators; and male:female rate-ratio computation	Methods - “Statistical Analyses” (Trend; Burden Decomposition; Temporal Decomposition; Sex-Disaggregated)
11	Describe how candidate models were evaluated and how final model(s) were selected	No new disease model is fitted; published GBD estimates are used, and their model selection (DisMod-MR 2.1 Bayesian meta-regression) is referenced. The deterministic two-component decomposition is fully specified and stated to be consistent with GBD analytical practice	Methods - “Temporal Decomposition”; GBD methods reference
12	Provide results of any model-performance evaluation and relevant sensitivity analyses	As a secondary analysis of published GBD outputs, no primary model fitting is performed; the decomposition is an exact, deterministic computation. The first-order nature of the decomposition and the contribution of population aging are addressed transparently	Methods - “Temporal Decomposition”; Discussion - limitations
13	Describe methods for calculating uncertainty; state which sources were and were not accounted for	All estimates are reported with 95% uncertainty intervals propagated from GBD’s 1,000-draw approach across modeling stages. Residual uncertainty (wide UIs) and the scope of uncertainty propagation are discussed	Methods - “Outcome Measures”; Discussion - limitations
14	State how analytic/statistical source code can be accessed	Software and packages are stated (Python 3.14.4: pandas, NumPy, matplotlib, seaborn; R 4.6: tidyverse, ggplot2, patchwork, ggrepel). The analysis code is available from the corresponding author on reasonable request	Methods - “Software”; “Data Availability Statement”
Results and discussion			
15	Provide published estimates in an efficiently extractable file format	Regional estimates are presented in Table 1 (age-standardized rates with 95% UIs, absolute DALYs, YLD proportions, and percent change for all 21 regions); full underlying estimates are extractable from the GHDx source	Results - Table 1; “Data Availability Statement”
16	Report a quantitative measure of the uncertainty of the estimates	95% uncertainty intervals are reported throughout the Results text and Table 1 (e.g., regional age-standardized DALY rates with 95% UIs)	Abstract; Results (all sections) (Table 1)
17	Interpret results in light of existing evidence; if updating prior estimates, explain reasons for changes	The Discussion situates findings against prior IBD/GBD studies and epidemiological-transition literature, comparing regional patterns, the YLL→YLD shift, sex differences, and MENA trends with published work	Discussion (throughout); References
18	Discuss limitations of the estimates, including modeling assumptions and data limitations	A dedicated limitations paragraph covers regional data-quality variation and underascertainment, residual uncertainty, regional-only resolution, CD-only scope, possible UC misclassification, the first-order decomposition, and potential COVID-19 effects	Discussion - “Strengths and limitations”
